# Risk Factors, Clinical Features, and Polygenic Risk Scores in Schizophrenia and Schizoaffective Disorder Depressive-Type

**DOI:** 10.1093/schbul/sbab036

**Published:** 2021-04-10

**Authors:** Charlotte A Dennison, Sophie E Legge, Leon Hubbard, Amy J Lynham, Stanley Zammit, Peter Holmans, Alastair G Cardno, Michael J Owen, Michael C O’Donovan, James T R Walters

**Affiliations:** 1 MRC Centre for Neuropsychiatric Genetics and Genomics, School of Medicine, Cardiff University, Cardiff, UK; 2 Centre for Academic Mental Health, Population Health Sciences, Bristol Medical School, University of Bristol, Bristol, UK; 3 Faculty of Medicine and Health, School of Medicine, University of Leeds, Leeds, UK

**Keywords:** polygenic risk score, depression, psychosis, diagnosis, phenotypes

## Abstract

There is controversy about the status of schizoaffective disorder depressive-type (SA-D), particularly whether it should be considered a form of schizophrenia or a distinct disorder. We aimed to determine whether individuals with SA-D differ from individuals with schizophrenia in terms of demographic, premorbid, and lifetime clinical characteristics, and genetic liability to schizophrenia, depression, and bipolar disorder. Participants were from the CardiffCOGS sample and met ICD-10 criteria for schizophrenia (*n* = 713) or SA-D (*n* = 151). Two samples, Cardiff Affected-sib (*n* = 354) and Cardiff F-series (*n* = 524), were used for replication. For all samples, phenotypic data were ascertained through structured interview, review of medical records, and an ICD-10 diagnosis made by trained researchers. Univariable and multivariable logistic regression models were used to compare individuals with schizophrenia and SA-D for demographic and clinical characteristics, and polygenic risk scores (PRS). In the CardiffCOGS, SA-D, compared to schizophrenia, was associated with female sex, childhood abuse, history of alcohol dependence, higher functioning Global Assessment Scale (GAS) score in worst episode of psychosis, lower functioning GAS score in worst episode of depression, and reduced lifetime severity of disorganized symptoms. Individuals with SA-D had higher depression PRS compared to those with schizophrenia. PRS for schizophrenia and bipolar disorder did not significantly differ between SA-D and schizophrenia. Compared to individuals with schizophrenia, individuals with SA-D had higher rates of environmental and genetic risk factors for depression and a similar genetic liability to schizophrenia. These findings are consistent with SA-D being a sub-type of schizophrenia resulting from elevated liability to both schizophrenia and depression.

## Introduction

Schizoaffective disorder depressive-type (SA-D) is characterized by the co-occurrence of depressive episodes with core features of schizophrenia.^1^ However, the validity of schizoaffective disorder, both depressive-type and bipolar-type (SA-BP), has long been debated,^2^ particularly given evidence of limited inter-rater reliability and low stability over time.^3,4^ Issues with reliability and stability may in part be driven by a lack of consensus on the definition of SA-D, particularly in terms of the duration and overlap of psychosis and depression. ICD-10 requires 2 weeks or more of psychosis, with psychosis and depression concurrent for at least part of the episode. DSM-IV^5^ requires psychosis for at least 1 month, including at least 2 weeks of hallucinations or delusions in the absence of any prominent symptoms of depression; depression must also occur for a substantial portion of the total illness. The major change in DSM-5^6^ was that depression was required to be present for the majority of the total duration of the lifespan of the illness (see table 1 for full criteria). As this criterion requires continuous observation over time, individuals can fluctuate between meeting and not meeting the diagnostic criteria, thus leading to an exaggerated perception of schizoaffective disorder as an unstable diagnosis.^7^

**Table 1.** Criteria for Schizoaffective Disorder Depressive-Type in ICD-10, DSM-IV, and DSM-5

	ICD-10	DSM-IV	DSM-5
Psychosis criteria	Symptoms from at least one group: a) Thought echo, insertion, withdrawal, or broadcasting b) Delusions of control, influence, or passivity c) Running commentary, third person voices, or voices coming from part of the body d) Bizarre or impossible delusions e) Grossly irrelevant or incoherent speech, or frequent use of neologisms f) Intermittent but frequent catatonic behavior	Two or more of the following: a) Delusions b) Hallucinations c) Disorganized speech d) Grossly disorganized or catatonic behavior e) Negative symptoms - Only one symptom is required if delusions are bizarre or hallucinations consist of running commentary or 2 or more voices conversing with each other.	Two or more of the following, at least one must be a), b), or c): a) Delusions b) Hallucinations c) Disorganized speech d) Grossly disorganized or catatonic behavior e) Negative symptoms
Depression criteria	A depressive episode of at least moderate severity.	A major depressive episode that must include depressed mood.	A major depressive episode that must include depressed mood.
Duration	- Psychosis criteria must be present for most of the time during a period of at least 2 wk. - Psychosis and depression must be met within the same episode, and concurrently for at least part of the episode.	- Uninterrupted period during which depression is concurrent with psychosis. - Psychosis criteria must be present for a significant portion of time during a 1-mo period. - Delusions or hallucinations for 2 or more weeks in the absence of mood during the lifetime duration of the illness. - Depression symptoms are present for a substantial portion of the total duration of the active and residual portions of the illness.	- Uninterrupted period during which depression is concurrent with psychosis. - Psychosis criteria must be present for a significant portion of time during a 1-mo period. - Delusions or hallucinations for 2 or more weeks in the absence of mood during the lifetime duration of the illness. - Depression symptoms are present for the majority of the total duration of the active and residual portions of the illness.

Research into schizoaffective disorder has often combined bipolar and depressive subtypes, despite evidence supporting their distinction.^8^ Studies that have done so have suggested that schizoaffective disorder may represent an intermediate category between schizophrenia and bipolar disorder. Individuals with schizoaffective disorder are more likely than individuals with schizophrenia to have been married, to have better cognitive functioning, and to have a better treatment response.^9^ Compared to individuals with mood disorders, individuals with schizoaffective disorder had poorer outcomes for these characteristics, as well as for various other demographics and clinical characteristics.^9^ Patterns of familial aggregation have also been reported to differ between those with schizophrenia, schizoaffective disorder, and bipolar disorder. In comparison with healthy controls, people with schizoaffective disorder have a higher family history of schizophrenia, schizoaffective disorder, and bipolar disorder,^10,11^ while diagnoses of schizophrenia or bipolar disorder are most strongly associated with a family history of schizophrenia or bipolar disorder, respectively.^11^ However, as noted above, much of this research has combined (or not distinguished between) bipolar and depressive subtypes of schizoaffective disorder, and therefore it is unclear whether these findings are applicable to both or only one subtype.

Studies that have distinguished SA-D from SA-BP have found that people with SA-D typically have poorer functioning than individuals with a diagnosis of psychotic depression, but better functioning than individuals with schizophrenia across a range of measures, including cognitive, premorbid, and overall functioning.^12,13^ In the context of shared symptoms, SA-D might be better considered an intermediate between schizophrenia and major depressive disorder^14^ rather than between schizophrenia and bipolar disorder. A twin study found that co-twins of individuals with SA-D were more likely to experience schizophrenia, SA-D, SA-BP, mania, and psychotic depression, than co-twins of unaffected controls.^8^ Therefore, SA-D may arise from elevated liability to schizophrenia and depression, and, to a lesser extent, mania.^15^ Family studies have supported this conclusion, showing that relatives of individuals with SA-D are at higher risk of schizophrenia, depression, and bipolar disorder,^16^ compared to relatives of unaffected individuals. Although SA-D, like schizophrenia and bipolar disorder, is highly heritable (around 80%),^17^ there has been very little genetic research into SA-D, individuals with SA-D being generally treated as if they have a diagnosis of schizophrenia.^18^

More research is needed to clarify the relationship between schizophrenia and SA-D in order to improve our understanding of the etiology of SA-D, and its relationships with other disorders, particularly schizophrenia and depression. Here, we aimed to establish whether individuals with SA-D differ from those with schizophrenia in terms of demographics, family history, premorbid factors, lifetime clinical characteristics, and genetic liability to schizophrenia, depression, and bipolar disorder.

## Methods

### Participants

Participants were drawn from CardiffCOGS,^19^ a cross-sectional study of individuals with a clinical diagnosis of schizophrenia or a related psychotic disorder. Individuals were recruited to CardiffCOGS from secondary psychiatric services between 2009 and 2017, completed a research interview based on the Schedules for Clinical Assessment in Neuropsychiatry (SCAN).^20^ Two additional samples were used for replication: the Cardiff Affected-sib and Cardiff F-series samples. Families were recruited from mental health services and relatives’ support groups to the Cardiff Affected-sib study^21^ from 1994–1997 where at least 2 affected siblings had a diagnosis of schizophrenia or a schizoaffective disorder. Affected sibling pairs where both individuals had a diagnosis of SA-BP were excluded. Unrelated individuals with a clinical diagnosis of schizophrenia or SA-D were recruited to the F-series study^22^ from 1997–2001, primarily through NHS secondary care services and additionally via an advertisement in a volunteer support organization. Participants in Cardiff Affected-sib and Cardiff F-series completed a research interview based on the SCAN or Present State Examination^23^ (PSE-9). Compared to the CardiffCOGS, the Cardiff Affected-sib and Cardiff F-series samples had a smaller number of phenotypic variables, and thus replication was restricted to those which were available across all 3 datasets. All studies have relevant NHS ethical approvals and all participants provided written informed consent.

For all 3 samples, trained researchers reviewed this information and completed lifetime assessments using the Operational Checklist Criteria for Psychotic Illness and Affective Illness (OPCRIT)^24^ and the SAPS and SANS,^25,26^ and reached a consensus diagnosis using ICD-10^1^ criteria. Individuals with a lifetime research diagnosis of ICD-10 schizophrenia or SA-D were included in the present study (table 2). Cohen’s kappa for inter-rater reliability for ICD-10 diagnoses within the Cardiff COGS sample was substantial (k = 0.76). Cardiff Affected-sib have previously reported an average κ score of 0.9 against consensus, indicating excellent reliability for diagnosis.^21^ Cardiff F-series previously reported a kappa >0.8 between raters for diagnosis.

**Table 2.** Number of Participants Included From Each Study With an ICD-10 Diagnosis of Schizophrenia and SA-D

Study	Schizophrenia	SA-D	Total
CardiffCOGS	713	151	864
Affected-sib	330	24	354
F-series	505	19	524
Total	1548	194	1724

Full definitions of all demographics, premorbid, and lifetime clinical characteristics are provided in supplementary table 1.

### Demographics, Family History, and Premorbid Characteristics

Details of demographics, family history, and premorbid characteristics were taken from the research interview, clinical case notes, and OPCRIT items. We obtained information on the following demographics: sex, family history of psychiatric illness, marital status, number of children, maximum educational attainment, and urbanicity, as well as on the following premorbid factors: premorbid social functioning, obstetric complications, premorbid IQ, and history of childhood physical or sexual abuse.^27^

### Lifetime Clinical Characteristics and Outcomes

Lifetime clinical characteristics were also derived from interview, clinical case notes, and OPCRIT items, and included course of disorder, mode of onset, antipsychotic response, MATRICS^28^ composite cognition, alcohol dependence, cannabis dependence, and other substance dependence. The following were included with respect to psychosis: age at onset of impairment, number of admissions, ever detained under the UK Mental Health Act 1983 for psychotic symptoms, number of episodes, and Global Assessment Scale (GAS) score regarding lowest level of functioning in worst episode. Measures of symptom severity were derived by combining the raw global scores from the lifetime most severe SAPS and SANS^25,26^ ratings (Legge et al., in submission). A positive symptoms score was calculated from the global hallucinations and global delusions scores; a disorganized symptoms score was calculated from global positive formal thought disorder and the inappropriate affect item; a diminished expressivity score was calculated from global affective flattening and global alogia; a reduced motivation and pleasure score was calculated from the global scores for avolition/apathy and anhedonia/asociality.

For individuals who had experienced at least one depressive episode under ICD-10 criteria, we included the following characteristics specific to the depressive episode: age at first impairment, age at first admission, ever admitted to hospital, longest episode duration, number of episodes, GAS score in worst episode, and whether depression onset occurred prior to psychosis onset.

### Genetic Data

The CardiffCOGS sample were genotyped in 2 waves on the Illumina HumanOmniExpressExome-8 and on the Illumina HumanOmniExpress-12. SNPs were excluded if: minor allele frequency (MAF) <0.01, genotyping rate <0.95, or Hardy-Weinberg equilibrium (HWE) *P*-value < 1 × 10^−6^. Samples missing >5% of genotypes were also excluded. Genotypes were imputed using the Haplotype Reference Consortium^29^ v1.1 reference panel. Best estimate genotype data were filtered to exclude by imputation quality score <0.8 and HWE *P* < value <1 × 10^−4^. PLINK v2.0^30^ was used to derive principal components for ancestry using SNPs with low levels of linkage disequilibrium, defined as *r*^2^ < 0.2, within 500kb windows. Principal components were used to restrict genetic analyses to individuals of European ancestry. Pairs of individuals with a kinship score >0.125 were identified and one member of each pair removed, preferentially retaining those with more complete phenotype data. Post-QC, genetic data were available for 692 individuals. Ten individuals were excluded due to ancestry restrictions, and 12 due to relatedness leaving 561 individuals with schizophrenia and 109 individuals with SA-D in the genetic liability analysis.

Genetic analyses within the Cardiff Affected-sib and Cardiff F-series samples is impaired due to the small number of individuals with SA-D, limited availability of high quality genetic data, and relatedness amongst the participants. Thus, polygenic analyses were restricted to the CardiffCOGS sample only.

### Polygenic Risk Scores

Polygenic risk scores (PRS) were derived using PRSice^31^ based on SNPs with INFO >0.9, MAF >0.10, and in relative linkage equilibrium (*i*^2^ <0.2 within 500kb windows), following criteria used by the Psychiatric Genomics Consortium.^18^ The extended major histocompatibility complex region (25–34Mb) was excluded due to its complex LD structure. PRS were calculated using the largest available genome-wide association summary statistics for schizophrenia,^32^ depression,^33^ and bipolar disorder^34^ at 6 thresholds: 5 × 10^−8^, 1 × 10^−4^, 0.001, 0.05, 0.1, and 0.5. Summary statistics used to define the association thresholds were generated after exclusion of the CardiffCOGS from the GWAS study. PRS were standardized as Z-scores. A pre-specified primary threshold of *P* < .05 for risk alleles for each disorder PRS was chosen given evidence that it is the optimal threshold for capturing schizophrenia liability.^18^ The other thresholds were also analyzed to ensure the results were not highly sensitive to our primary test threshold.

## Analysis

### Univariable Analysis

Primary analyses were conducted within the CardiffCOGS sample. The continuous variables were standardized as Z-scores to allow for comparison across characteristics. Primary analyses involved testing for differences between schizophrenia and SA-D via logistic regression with respect to individual demographics and clinical characteristics. As we found a significant association between sex and diagnosis, all other analyses were covaried for sex, as well as age at interview. Depression characteristics were analyzed only in those with at least one depressive episode (*N* = 426 with schizophrenia, *n* = 151 with SA-D).

### Replication

The demographic and clinical characteristics that were significantly associated in the primary univariable analysis (*P* < .05) and that were available in both the Cardiff Affected-sib and Cardiff F-series samples were tested for replication (see supplementary table 2 for characteristics included). A meta-analysis was conducted of the results from the Cardiff Affected-sib and Cardiff F-series samples, using a fixed effect model weighted by standard error, conducted in the R package *meta*.^35^

### Multivariable Analysis

For variables that were associated with SA-D (*P* < 0.05) in the univariable analyses, we performed multivariable analysis to clarify whether the associations were independent. Characteristics were grouped into 4 categories which were analyzed separately: demographics and premorbid; psychosis; depression; other clinical, (see supplementary table 7 for groupings of characteristics). Each regression model contained sex and age at interview as covariates, with diagnosis as the outcome.

### Polygenic Risk Scores

Polygenic risk scores (PRS) were analyzed using logistic regressions, with principal components 1 to 5 and sex included as covariates.

## Results

### Demographics, Family History, and Premorbid Characteristics

**Fig. 1.**
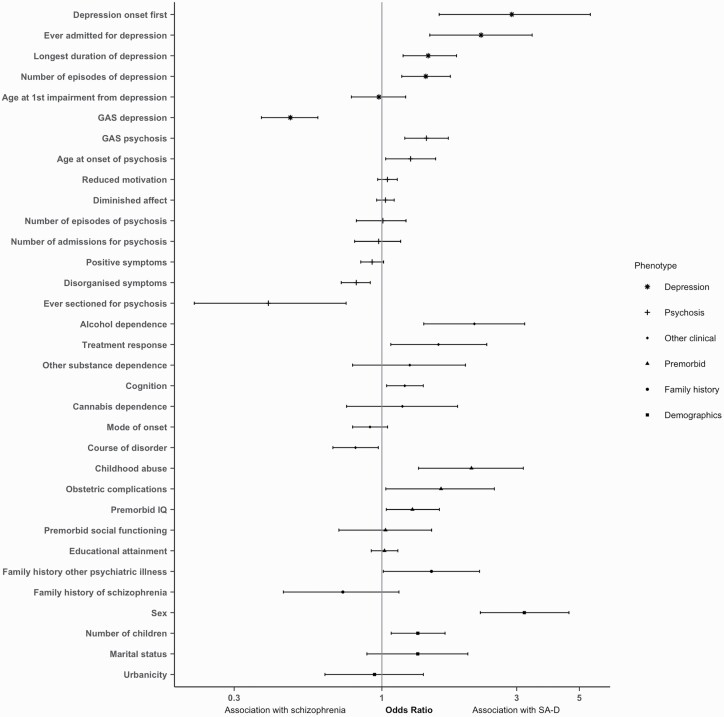
Odds ratios (OR) and confidence interval for each characteristic in the Cardiff COGS sample. OR > 1 indicates association with SA-D; OR < 1 indicates association with schizophrenia. Clinical characteristics of depression are analyzed only in participants with at least one episode of depression.

In the univariable analyses, compared to schizophrenia, SA-D was associated with female sex (OR = 3.19, 95% confidence intervals [CI] = 2.23–4.59, *P* = 2.8 × 10^−10^), self-reported experience of childhood abuse (OR = 2.07, CI = 1.35–3.17, *P* =7.9 × 10^−4^), obstetric complications (OR = 1.62, CI = 1.03–2.50, *P* = .03), and family history of psychiatric disorder other than schizophrenia (OR = 1.50, CI = 1.01–2.22, *P* = .04) but not family history of schizophrenia. Those with SA-D had a greater number of children (OR = 1.34, CI = 1.08–1.67, *P* = 0.01), and higher premorbid IQ (OR = 1.28, 95%CI= 1.04–1.60, *P* = 0.02) than individuals with schizophrenia (figure 1 and supplementary table 3).

### Lifetime Clinical Characteristics

Compared to schizophrenia, SA-D was associated with alcohol dependence (OR = 2.12, CI=1.41–3.20, *P* = 3.2 × 10^−4^), positive antipsychotic response (OR = 1.59, CI = 1.08–2.35, *P* = .02), better current cognitive functioning (OR = 1.20, CI = 1.04–1.40, *P* = .01), and a less chronic course of disorder (OR = 0.81, CI = 0.67–0.97, *P* = .02). SA-D was also associated with milder psychosis related phenotypes, namely better functioning GAS score in worst episode of psychosis (OR = 1.44, CI = 1.20–1.72, *P* = 5.8 × 10^−5^), older age at onset of psychosis (OR = 1.26, CI = 1.03–1.54, *P* = .02), reduced severity of disorganized symptoms (OR = 0.81, CI = 0.72–0.91, *P* = 5.3 × 10^−4^), and a lower risk for being detained under the mental health act for psychosis (OR = 0.40, CI = 0.22–0.75, *P* = 3.2 × 10^−3^) (figure 1 and supplementary table 3).

To investigate differences in depression symptoms and outcomes between those with SA-D and schizophrenia, we restricted the schizophrenia sample to those who had experienced at least one episode of ICD-10 defined depression. SA-D was strongly associated with the onset of depression occurring prior to psychosis onset (OR=2.88, CI=1.59–5.47, *P* = 7.1 × 10^−4^) and a greater likelihood of an admission for depression (OR=2.24, CI=1.48–3.40, *P* = 1.4 × 10^−4^). SA-D was also associated with longer duration of depression (OR = 1.46, CI = 1.19–1.84, *P* = 6.0 × 10^−4^), more episodes of depression (OR = 1.43, CI = 1.18–1.75, *P* = 3.7 × 10^−4^), and lower functioning GAS score in worst episode of depression (OR = 0.47, CI = 0.37–0.59, *P* = 2.0 × 10^−10^) (figure 1 and supplementary table 3).

Full results are presented in supplementary table 3. As sex was the strongest predictor of diagnosis, we looked for sex-specific effects across all lifetime demographic and clinical characteristics but the results were consistent between females and males (supplementary figure 1). Univariable analyses were repeated using DSM-IV defined schizophrenia and SA-D, and effect sizes were consistent between ICD-10 and DSM-IV for all characteristics (supplementary table 4). Additionally, we tested for differences between individuals with SA-D and SA-BP. We note the since the SA-BD sample is very small (*N* = 104 individuals with ICD-10 SA-BP), and follow up samples were not available, we consider the results as provisional (supplementary table 5).

### Replication

**Fig. 2.**
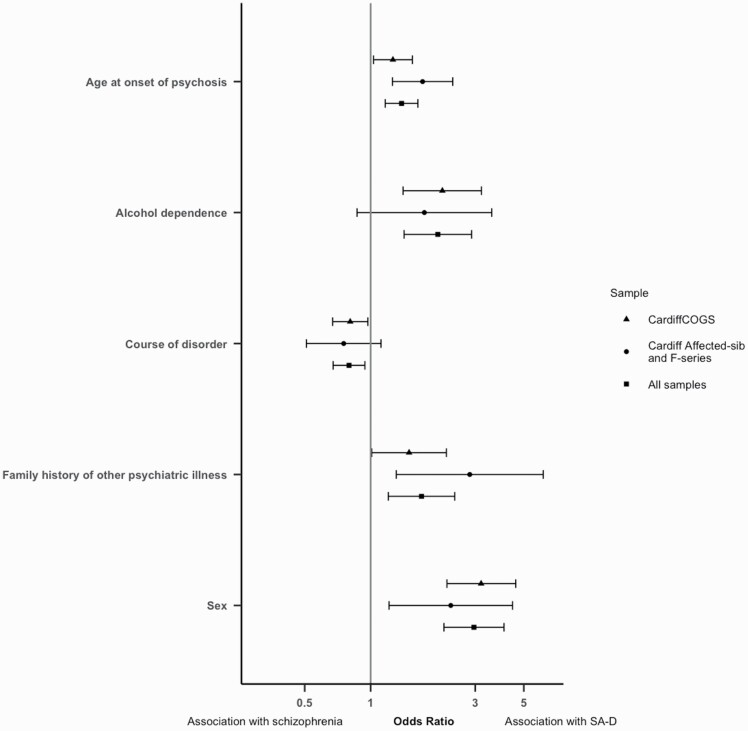
Odds ratios (OR) and confidence interval for each lifetime clinical characteristic included in the replication and meta-analysis, analyzed in the CardiffCOGS sample, the replication samples, or in the meta-analysis of all samples. OR > 1 indicates association with SA-D; OR<1 indicates association with schizophrenia.

Five of the variables associated with SA-D in CardiffCOGS were present in the Cardiff Affected-sib and Cardiff F-series samples. In the replication samples all 5 of these variables were associated with SA-D with the same direction of effect as the original associations, with 3 of these reaching significance. SA-D was significantly associated with female sex (OR = 2.32, CI = 1.21–4.44, *P =* .01), family history of psychiatric disorder other than schizophrenia (OR = 2.83, CI = 1.31–6.13, *P* = .01), and older age at onset of psychosis (OR = 1.73, CI = 1.26–2.37, *P* = 7.4 × 10^−4^) (supplementary table 6 and figure 2). When all 3 samples were meta-analyzed, SA-D was significantly associated with all 5 characteristics (figure 2).

### Multivariable Models

In the demographics and premorbid model, SA-D remained significantly associated with female sex (OR = 2.22, CI = 1.14–4.36, *P* = .02), and experience of childhood abuse (OR = 2.80, CI = 1.36–5.71, *P* = 4.6 × 10^−3^), but not with family history of psychiatric illness, number of children, obstetric complications, premorbid IQ, or age at onset of psychosis. In the clinical characteristics model, greater alcohol dependence was significantly associated with SA-D (OR = 2.08, CI = 1.31–3.28, *P* = 1.8 × 10^−3^), but not with course of disorder, cognition, or antipsychotic response. In the psychosis model, SA-D was significantly associated with better functioning GAS score in worst episode of psychosis (OR = 1.31, CI = 1.07–1.61, *P* = .01) and reduced lifetime severity of disorganized symptoms (OR = 0.84, CI = 0.74–0.96, *P* = .01), but not with being detained under the mental health act for psychosis. In the depression model, SA-D was associated with a greater number of episodes of depression (OR = 1.62, CI = 1.14–2.32, *P* = .01), and lower functioning GAS score in worst episode of depression (OR = 0.49, CI = 0.33–0.72, *P* = 4.8 × 10^−4^), but not with ever being admitted for depression, longest duration of depression, or having depression onset prior to psychosis onset. Full results for the multivariable models are presented in supplementary table 7.

In order to better compare the univariable and multivariable models, we repeated the significant univariable analyses restricted to individuals who had complete data for the variables entered into the appropriate multivariable model. Effect sizes remained consistent between the restricted and full sample analyses and are presented in supplementary table 7.

### Polygenic Risk Scores

**Fig. 3.**
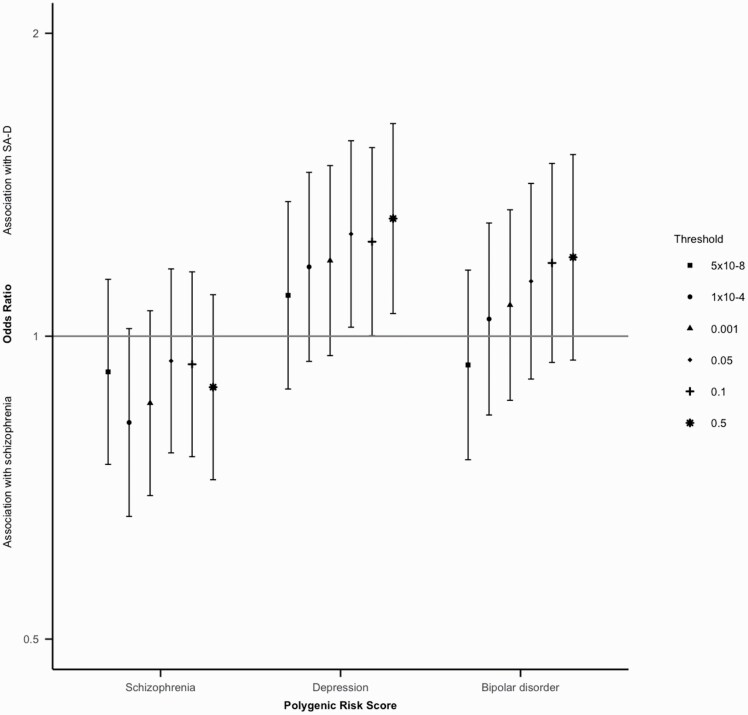
Odds ratios (OR) and 95% confidence intervals for all thresholds tested for schizophrenia, depression and bipolar disorder polygenic risk scores in the Cardiff COGS sample. OR > 1 indicated association with SA-D; OR < 1 indicates association with schizophrenia.

At the primary p-value threshold of p<0.05, schizophrenia PRS was not significantly different between individuals with SA-D and with schizophrenia (OR = 0.94, CI = 0.77–1.17, *P* = .60). Bipolar disorder PRS was also not significantly different between individuals with SA-D and with schizophrenia (OR = 1.13, CI = 0.91–1.42), *P* = .27). Depression PRS was significantly higher in individuals with SA-D compared to individuals with schizophrenia (OR = 1.26, CI = 1.02–1.56, *P* = .03) (figure 3). These results were broadly consistent across *P* -value thresholds (supplementary table 8).

## Discussion

We present novel evidence to show that SA-D differs from schizophrenia in terms of demographics, clinical course, outcomes, and genetic liability to depression, but not genetic liability to schizophrenia.

### Greater Environmental and Genetic Risk for Depression in SA-D

Several risk factors for depression occurred more frequently in people with SA-D, compared with those with schizophrenia, including female sex, experience of childhood physical and/or sexual abuse, and alcohol dependence.^36–38^ Previous twin research has suggested that individuals with SA-D may have an elevated genetic liability to depression^8^; we support this with novel evidence that PRS for depression is elevated in individuals with SA-D compared to those with schizophrenia, who themselves are known to have elevated genetic liability to depression compared with controls.^39^ Family history of psychiatric disorders, other than schizophrenia, was also associated with SA-D in the univariable analysis and replicated in additional samples, further suggesting that individuals with SA-D may have an elevated genetic liability to other disorders. Individuals with schizophrenia and SA-D did not significantly differ in terms of genetic liability to schizophrenia or environmental risk factors for schizophrenia, including urbanicity, poor premorbid social functioning, and cannabis dependence.^40^ Thus, SA-D may represent a subset of people with schizophrenia but who have an additional burden of environmental and genetic risk factors for depression.

### Greater Severity of Depression

Prominence of depressive episodes in the clinical picture is a criterion for SA-D, suggesting that individuals with SA-D should experience more severe depression than individuals with schizophrenia. Our univariable analyses support this, while the multivariable analysis of depression characteristics found that the number of episodes of depression and the functioning in the worst episode of depression were the variables most strongly associated with SA-D. As our analysis of depression characteristics was restricted to individuals who had experienced at least one episode of depression, the associations with depression characteristics are not due to the presence of individuals with schizophrenia without comorbid depression.

### Reduced Burden of Psychosis

Individuals with SA-D had better psychosis related outcomes, predominantly characterized in the multivariable analysis by better functioning in the worst episode of psychosis and reduced lifetime severity of disorganized symptoms. These findings are consistent with a number of studies showing reduced severity of psychotic symptoms in schizoaffective disorders compared to schizophrenia,^9,41^ although those studies did not stratify by subtype of schizoaffective disorder. Despite the clinical characteristics suggesting a less severe psychosis phenotype in those with SA-D, schizophrenia polygenic risk score did not significantly differentiate between SA-D and schizophrenia. Previous research has found that schizophrenia PRS is not significantly associated with psychosis related variables, such as positive symptom severity^42^ (Legge et al., in submission), in people with schizophrenia. In this respect, schizophrenia PRS does not seem to relate to psychosis severity measures or outcomes in those with schizophrenia or SA-D.

### Neurodevelopmental Spectrum

It has been postulated that schizophrenia lies on a spectrum of neurodevelopmental disorders, with intellectual disability and autism spectrum disorders having a greater neurodevelopmental component than schizophrenia, and affective disorders having less.^43^ The results of our univariable analysis, particularly with regard to female sex, better current cognitive functioning, less chronic course of disorder, older age at onset of psychosis, and greater burden of affective symptoms, suggests that SA-D may occupy a less severe position on this proposed spectrum than schizophrenia.^44^ Whilst not all of these neurodevelopmental risk factors were associated in the multivariable models, we note that the effect sizes of these variables are comparable to those found in the univariable models.

Previous research that did not distinguish between SA-BP and SA-D has shown mixed findings when comparing schizoaffective disorders to schizophrenia. Some studies reported a higher proportion of women with schizoaffective disorder compared to schizophrenia,^9,45^ which we found to be one of the most substantial differences between individuals with SA-D and schizophrenia. At a population level, women are more likely to experience depression than men,^36^ and it is possible that this may also be the case for depression comorbid with other primary psychiatric diagnoses.

### Limitations

Our study is limited by the relatively small number of individuals with SA-D, and although we have replicated some of the key findings, validation in other samples is necessary to examine whether our findings are fully robust to, for example, unknown biases in ascertainment. Whilst we did not detect any between group differences in schizophrenia or bipolar disorder PRS, given our relatively small target sample there could be differences between the group that we are not powered to detect. The proportion of individuals with SA-D differed across the samples, with the Cardiff Affected-sib and Cardiff F-series samples containing fewer individuals with SA-D compared to schizophrenia than was seen in the CardiffCOGS. This is likely to be because depression data for participants in the CardiffCOGS was derived from both clinical records and from direct questions about depressive symptoms and episodes as part of the SCAN interview, whereas participants in Cardiff Affected-sib and Cardiff F-series were not directly asked these questions, but this data was drawn from clinical notes.

## Conclusions

Compared to schizophrenia, SA-D was associated with several risk factors for depression, including female sex and greater alcohol dependence. We observed an increased burden of depression in individuals with SA-D, including higher polygenic risk score for depression, compared to individuals with schizophrenia. Our results are consistent with SA-D being a form of schizophrenia which is modified by an elevated liability to depression.

## Funding

This work was supported by the Medical Research Council (MC_PC_17212, G0800509, and MR/L010305/1); and the National Institute of Mental Health (1U01MH109514-01).

## Supplementary Material

sbab036_suppl_Supplementary_Material-Figures

sbab036_suppl_Supplementary-Material-Tables
