# Supplementary material for: Risk Factors, Clinical Features, and Polygenic Risk Scores in Schizophrenia and Schizoaffective Disorder Depressive-Type
Source: Schizophr Bull. 2021 Apr 10;47(5):1375–84. doi: 10.1093/schbul/sbab036 (PMC8379553; doi:10.1093/schbul/sbab036)

## Supplementary Figure 1. Demographic, premorbid, and lifetime clinical characteristic results separated by sex.

Odds ratios and confidence interval for each lifetime clinical characteristic in the Cardiff COGS sample, separated into female-only, male-only, and the mixed sex analyses. Odds ratio >1 indicates association with SA-D; odds ratio <1 indicates association with schizophrenia. Clinical characteristics of depression are analysed only in participants with at least one episode of major depression.


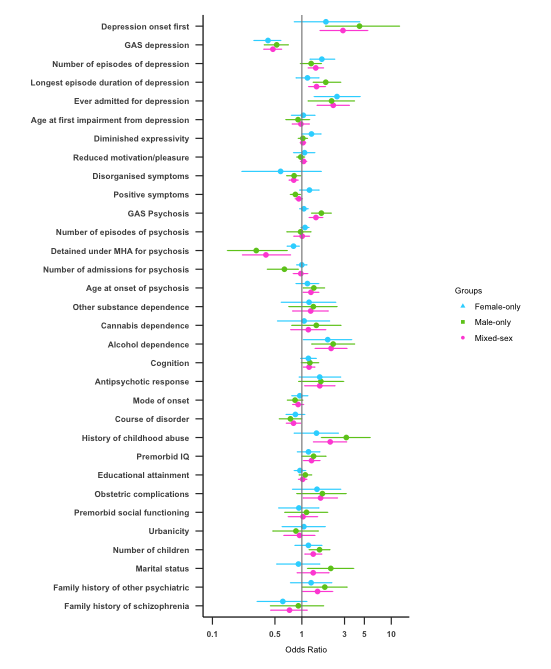

Supplement: sbab036_suppl_Supplementary_Material-Figures [file sbab036_suppl_supplementary_material-figures.docx]
