# Supplementary material for: Risk Factors, Clinical Features, and Polygenic Risk Scores in Schizophrenia and Schizoaffective Disorder Depressive-Type
Source: Schizophr Bull. 2021 Apr 10;47(5):1375–84. doi: 10.1093/schbul/sbab036 (PMC8379553; doi:10.1093/schbul/sbab036)
Supplement: sbab036_suppl_Supplementary-Material-Tables [file sbab036_suppl_supplementary-material-tables.docx]

Table of Contents

[Supplementary Tables 2](#_Toc66456091)

[Supplementary Table 1. Variable definitions. 2](#_Toc66456092)

[Supplementary Table 2. Characteristics included in the replication meta-analysis. 5](#_Toc66456093)

[Supplementary Table 3. Demographic, premorbid, and lifetime clinical characteristics in Cardiff COGS. 6](#_Toc66456094)

[Supplementary Table 4. Comparison of demographic, premorbid, and lifetime clinical characteristic results using ICD-10 and DSM-IV defined diagnosis. 8](#_Toc66456095)

[Supplementary Table 5 Comparison of demographic, premorbid, and lifetime clinical characteristic results between schizoaffective bipolar-type and depressive-type. 10](#_Toc66456096)

[Supplementary Table 6. Results of replication and meta-analysis. 11](#_Toc66456097)

[Supplementary Table 7. Demographic and clinical characteristics in restricted sample univariable and multivariable models. 12](#_Toc66456098)

[Supplementary Table 8. Polygenic risk score results. 13](#_Toc66456099)

# Supplementary Tables

## Supplementary Table 1. Variable definitions.

Definition of each clinical characteristic and number included from CardiffCOGS sample.

| Variable | Descriptions | | N (864) |
| --- | --- | --- | --- |
| *Demographics and family history* | |  |  |
| Sex | Self-reported sex. 1 = male, 2 = female. | | 864 (100%) |
| Family history of schizophrenia | OPCRIT item 13  Family history of schizophrenia in a first or second degree relative. | | 739 (86%) |
| Family history of other psychiatric illness | OPCRIT item 14  First or second degree relative with psychiatric disorder severe enough to warrant psychiatric referral, excluding schizophrenia | | 735 (85%) |
| Marital history | Ever been married. 0 = no, 1= yes. | | 842 (97%) |
| Number of children | Number of children. | | 491 (57%) |
| Educational attainment | Highest educational attainment: 0 = none, 1 = 11+, 2 = CSE, 3 = O-Level or GCSE, 4 = A-level, 5 = Degree | | 840 (97%) |
| Urbanicity | Main place of upbringing. 0 = village or town, 1 = city | | 714 (83%) |
| *Premorbid functioning* |  | |  |
| Premorbid social functioning | OPCRIT item 10: Patient found difficulty entering or maintaining normal social relationships, showed persistent social isolation, withdrawal or maintained solitary interests prior to onset of psychotic symptoms. | | 821 (95%) |
| Obstetric complications | Complication with the participant’s birth such as low birth weight, hypoxia or assisted delivery, and/or complication with their mother’s pregnancy such as prematurity, pre-eclampsia or placental problems. | | 860 (99%) |
| Premorbid IQ | Premorbid IQ estimated from the National Adult Reading Test (NART). Predicted WAIS-R full scale IQ = 130.6-1.24*NART error score. | | 696 (82%) |
| Childhood physical or sexual abuse | Childhood physical or sexual abuse reported in the Childhood Life Events Questionnaire^1^ (CLEQ) delivered at interview. Participants were not explicitly asked about abuse, but were asked “are there any other significant life events you experienced as a child that are not mentioned above”. | | 795 (92%) |
| *Lifetime clinical characteristics* | | |  |
| Course of disorder | OPCRIT item 90. 1 = single episode with good recovery, 2 = multiple episodes with good recovery between, 3 = multiple episodes with partial recovery between, 4 = continuous chronic illness, 5 = continuous chronic illness with deterioration | | 847 (98%) |
| Mode of onset | OPCRIT item 5. 1 = abrupt onset definable to within hours or up to three days, 2 = acute onset definable to within one week, 3 = moderately acute onset definable within one month, 4 = gradual onset over a period up to six months, 5 = Insidious onset over period greater than six months | | 686 (79%) |
| Antipsychotic response | OPCRIT item 89. 1 = Substantial improvement in psychotic symptoms either subjectively or according to medical records, or if relapse occurs when medication is stopped. Rated 0 if patient did not meet these criteria or was treated with Clozapine for treatment resistance. | | 809 (94%) |
| Cognition | Full scale composite cognition score as measured by the MATRICS Consensus Cognitive Battery, imputed and standardised into z-scores. | | 809 (94%) |
| Alcohol dependence | OPCRIT item 78. One of the following must have occurred persistently for at least one month: Continued use despite knowledge of having a persistent or recurrent social, occupational, psychological or physical problem that is caused or exacerbated by alcohol; or recurrent use in situations in which it is physically hazardous; or symptoms definitely indicative of dependence. | | 775 (90%) |
| Cannabis dependence | OPCRIT item 79. One of the following must have occurred persistently for at least one month: continued use despite knowledge of having a persistent or recurrent social, occupational, psychological or physical problem that is caused or exacerbated by cannabis; or recurrent use in situations in which it is physically hazardous; or symptoms definitely indicative of dependence. | | 790 (91%) |
| Other substance dependence | OPCRIT item 80. One of the following must have occurred persistently for at least one month: Continued use despite knowledge of having a persistent or recurrent social, occupational, psychological or physical problem that is caused or exacerbated by substance use; or recurrent use in situations in which it is physically hazardous; or symptoms definitely indicative of dependence. | | 811 (94%) |
| *Psychosis* |  | |  |
| Age of onset | OPCRIT item 4. The age at which treatment was first sought or if earlier when symptoms caused significant impairment. | | 829 (96%) |
| Number of admissions | Total number of psychiatric hospital admissions for psychosis, including inpatient, day hospital and intensive home treatment by the crisis team. | | 829 (96%) |
| Ever detained under the mental health act | Ever detained under section 2 or 3 of the Mental Health Act for psychosis. | | 744 (86%) |
| Number of episodes | Number of episodes of psychosis. | | 748 (87%) |
| Global assessment scale score in worst episode of psychosis | Lifetime worst Global Assessment Scale (GAS) score in a psychotic episode. Higher score indicates better functioning. | | 847 (88%) |
| Positive symptoms | Lifetime severity of positive symptoms, on a scale of 0 – 10. Derived from SAPS Global Hallucinations and SAPS Global Delusions | |  |
| Disorganised symptoms | Lifetime severity of disorganised symptoms, on a scale of 0 – 10. Derived from SAPS Global Positive Thought Disorder and SANS Inappropriate Affect | |  |
| Diminished expressivity | Lifetime severity of negative symptoms of diminished expressivity, on a scale of 0 - 10. Derived from SANS Global Affective Flattening and SANS Global Alogia. | |  |
| Reduced motivation and pleasure | Lifetime severity of negative symptoms of motivation and pleasure, on a scale of 0 – 10. Derived from SANS Global Anhedonia and SANS Global Avolition/Apathy. | |  |
| *Depression*  NB: only participants with at least one episode of major depression are included in these categories. | | | |
| Age at first impairment | Age at which depressive episode caused significant impairment, such as received treatment, disruption to work or school, police involvement, psychotic features, or family split up. | | 517 (60%) |
| Ever admitted to hospital | Ever admitted to psychiatric hospital for depression, including inpatient, day hospital and intensive home treatment by the crisis team. | | 577 (67%) |
| Longest episode duration | Duration of the longest episode of depression meeting ICD-10 diagnostic criteria for major depressive episode. | | 454 (53%) |
| Number of episodes | Number of episodes of depression that met ICD-10 diagnostic criteria for major depressive episode. | | 575 (67%) |
| Global assessment scale score in worst episode of depression | Lifetime worst GAS score in a depressive episode. Higher score indicates better functioning. | | 549 (64%) |
| Depression onset first | Onset of depression occurred prior to onset of psychosis. | | 500 (58%) |

## Supplementary Table 2. Characteristics included in the replication meta-analysis.

Columns indicate characteristics, and total number of individuals with data from the replication datasets with a diagnosis of schizophrenia or SA-D.

| Variable | SCZ  Total N | SA-D  Total N |
| --- | --- | --- |
| Sex | 835 | 43 |
| Family history of other psychiatric disorder | 792 | 42 |
| Course of disorder | 679 | 32 |
| Alcohol dependence | 791 | 42 |
| Age at onset of psychosis | 811 | 40 |

## Supplementary Table 3. Demographic, premorbid, and lifetime clinical characteristics in Cardiff COGS.

N (%) indicates number and percentage of individuals within the diagnostic group that positively report the clinical characteristic for binary traits. For continuous traits, the mean and standard deviation are reported. Odds ratio with 95% confidence intervals and p-value are reported for the association between each phenotype and SA-D in the primary univariable analysis.

| **Phenotype** | **SCZ** | **SA-D** | **OR (95% CI)** | **P-value** |
| --- | --- | --- | --- | --- |
|  | **N (%) / Mean (SD)** | **N (%) / Mean (SD)** |  |  |
| Female sex | 213 (29.87%) | 87 (57.62%) | 3.19 (2.23 – 4.59) | 2.8x10^-10^ |
| Family history of schizophrenia | 152 (25%) | 28 (21.37%) | 0.73 (0.45 – 1.15) | 0.18 |
| Family history of other psychiatric illness | 224 (37.27%) | 68 (50.75%) | 1.50 (1.01 – 2.22) | 0.04 |
| Marital history | 185 (26.62%) | 55 (37.41%) | 1.34 (0.88 – 2.01) | 0.16 |
| Number of children | 0.67 (1.26) | 1.27 (1.47) | 1.34 (1.08 – 1.67) | 0.01 |
| Urbanicity | 243 (41.68%) | 51 (38.93%) | 0.94 (0.63 – 1.40) | 0.77 |
| Premorbid social functioning | 277 (40.92%) | 63 (43.75%) | 1.03 (0.70 – 1.50) | 0.88 |
| Obstetric complications | 118 (16.62%) | 36 (24%) | 1.62 (1.03 – 2.50) | 0.03 |
| Educational attainment | 2.59 (1.7) | 2.62 (1.73) | 1.02 (0.92 – 1.14) | 0.70 |
| Premorbid IQ | 104.67 (10.32) | 106.7 (9.29) | 1.28 (1.04 – 1.60) | 0.02 |
| Childhood abuse | 112 (17.02%) | 46 (33.58%) | 2.07 (1.35 – 3.17) | 7.9x10^-4^ |
| Course of disorder | 3.45 (0.94) | 3.22 (0.96) | 0.81 (0.67 – 0.97) | 0.02 |
| Mode of onset | 3.68 (1.4) | 3.48 (1.42) | 0.91 (0.79 – 1.05) | 0.18 |
| Antipsychotic response | 312 (46.15%) | 78 (58.65%) | 1.59 (1.08 – 2.35) | 0.02 |
| Cognition | -2.42 (1.35) | -2.14 (1.44) | 1.20 (1.04 – 1.40) | 0.01 |
| Alcohol dependence | 166 (25.98%) | 51 (37.5%) | 2.12 (1.41 – 3.20) | 3.2x10^-4^ |
| Cannabis dependence | 232 (35.75%) | 42 (29.79%) | 1.18 (0.75 – 1.85) | 0.47 |
| Other substance dependence | 172 (25.83%) | 35 (24.14%) | 1.26 (0.79 – 1.97) | 0.33 |
| Age at onset of psychosis | 24.1 (8.61) | 26.41 (9.54) | 1.26 (1.03 – 1.54) | 0.02 |
| Number admissions for psychosis | 4.66 (5.13) | 4.68 (4.77) | 0.97 (0.80 – 1.17) | 0.78 |
| Detained under the mental health act for psychosis | 570 (93.14%) | 114 (86.36%) | 0.40 (0.22 – 0.75) | 3.2x10^-3^ |
| Number of episodes of psychosis | 5.94 (7.54) | 6.05 (7.72) | 1.01 (0.81 – 1.22) | 0.94 |
| GAS psychosis | 19.51 (6.78) | 22.11 (7.68) | 1.44 (1.20 – 1.72) | 5.8x10^-5^ |
| Positive symptoms | 6.29 (1.95) | 6.06 (1.8) | 0.92 (0.84 – 1.01) | 0.09 |
| Disorganised symptoms | 1.56 (1.84) | 0.98 (1.65) | 0.81 (0.72 – 0.91) | 5.3x10^-4^ |
| Diminished expressivity | 2.99 (2.63) | 3.02 (2.51) | 1.03 (0.96 – 1.10) | 0.27 |
| Reduced motivation/pleasure | 4.55 (2.29) | 4.8 (2.41) | 1.05 (0.97 – 1.13) | 0.42 |
| Age at first impairment from depression | 22.88 (8.76) | 22.62 (8.35) | 0.98 (0.78 – 1.21) | 0.83 |
| Ever admitted for depression | 90 (21.13%) | 59 (39.07%) | 2.24 (1.48 – 3.40) | 1.4x10^-4^ |
| Longest duration of depression | 39.81 (69.99) | 75.29 (118.12) | 1.46 (1.19 – 1.84) | 6.0x10^-4^ |
| Number of episodes of depression | 6.47 (7.65) | 9.92 (8.98) | 1.43 (1.18 – 1.75) | 3.7x10^-4^ |
| GAS depression | 32.64 (10.75) | 25.17 (8.58) | 0.47 (0.37 – 0.59) | 2.0x10^-10^ |
| Depression onset first | 145 (60.92%) | 78 (82.98%) | 2.88 (1.59 – 5.47) | 7.1x10^-4^ |

## Supplementary Table 4. Comparison of demographic, premorbid, and lifetime clinical characteristic results using ICD-10 and DSM-IV defined diagnosis.

Odds ratio with 95% confidence intervals and p-value are reported for the association between each phenotype and SA-D when diagnosis was defined according to either ICD-10 or DSM-IV criteria.

| **Phenotype** | **ICD-10** | | **DSM-IV** | |
| --- | --- | --- | --- | --- |
|  | **OR (95% CI)** | **P-value** | **OR (95% CI)** | **P-value** |
| Female sex | 3.19 (2.23 – 4.59) | 2.8x10^-10^ | 3.21 (2.28 - 4.53) | 2.7x10^-11^ |
| Family history of schizophrenia | 0.73 (0.45 – 1.15) | 0.18 | 0.64 (0.40 - 1.01) | 0.06 |
| Family history of other psychiatric illness | 1.50 (1.01 – 2.22) | 0.04 | 1.63 (1.12 - 2.37) | 0.01 |
| Marital history | 1.34 (0.88 – 2.01) | 0.16 | 1.47 (1.00 - 2.17) | 0.05 |
| Number of children | 1.34 (1.08 – 1.67) | 0.01 | 1.23 (1.00 - 1.52) | 0.04 |
| Urbanicity | 0.94 (0.63 – 1.40) | 0.77 | 0.84 (0.58 - 1.23) | 0.38 |
| Premorbid social functioning | 1.03 (0.70 – 1.50) | 0.88 | 1.03 (0.71 - 1.47) | 0.88 |
| Obstetric complications | 1.62 (1.03 – 2.50) | 0.03 | 1.52 (0.98 - 2.31) | 0.05 |
| Educational attainment | 1.02 (0.92 – 1.14) | 0.70 | 1.02 (0.92 - 1.13) | 0.70 |
| Premorbid IQ | 1.28 (1.04 – 1.60) | 0.02 | 1.25 (1.02 - 1.53) | 0.04 |
| Childhood abuse | 2.07 (1.35 – 3.17) | 7.9x10^-4^ | 2.18 (1.45 - 3.25) | 1.5x10^-4^ |
| Course of disorder | 0.81 (0.67 – 0.97) | 0.02 | 0.78 (0.66 - 0.93) | 0.01 |
| Mode of onset | 0.91 (0.79 – 1.05) | 0.18 | 0.97 (0.85 - 1.12) | 0.68 |
| Antipsychotic response | 1.59 (1.08 – 2.35) | 0.02 | 1.68 (1.16 - 2.45) | 0.01 |
| Cognition | 1.20 (1.04 – 1.40) | 0.01 | 1.17 (1.02 - 1.35) | 0.03 |
| Alcohol dependence | 2.12 (1.41 – 3.20) | 3.2x10^-4^ | 1.82 (1.23 - 2.69) | 2.7x10^-3^ |
| Cannabis dependence | 1.18 (0.75 – 1.85) | 0.47 | 1.15 (0.75 - 1.76) | 0.51 |
| Other substance dependence | 1.26 (0.79 – 1.97) | 0.33 | 1.14 (0.73 - 1.76) | 0.57 |
| Age at onset of psychosis | 1.26 (1.03 – 1.54) | 0.02 | 1.31 (1.08 - 1.59) | 0.01 |
| Number admissions for psychosis | 0.97 (0.80 – 1.17) | 0.78 | 0.94 (0.78 - 1.12) | 0.52 |
| Detained under the mental health act for psychosis | 0.40 (0.22 – 0.75) | 3.2x10^-3^ | 0.41 (0.23 - 0.73) | 1.8x10^-3^ |
| Number of episodes of psychosis | 1.01 (0.81 – 1.22) | 0.94 | 0.98 (0.79 - 1.18) | 0.86 |
| GAS psychosis | 1.44 (1.20 – 1.72) | 5.8x10^-5^ | 1.49 (1.26 - 1.76) | 3.8x10^-6^ |
| Positive symptoms | 0.92 (0.84 – 1.01) | 0.09 | 0.91 (0.84 - 0.99) | 0.04 |
| Disorganised symptoms | 0.81 (0.72 – 0.91) | 5.3x10^-4^ | 0.79 (0.70 - 0.89) | 9.3x10^-5^ |
| Diminished expressivity | 1.03 (0.96 – 1.10) | 0.27 | 1.07 (0.99 - 1.16) | 0.07 |
| Reduced motivation/pleasure | 1.05 (0.97 – 1.13) | 0.42 | 1.04 (0.97 - 1.11) | 0.25 |
| Age at first impairment from depression | 0.98 (0.78 – 1.21) | 0.83 | 1.00 (0.80 - 1.23) | 0.97 |
| Ever admitted for depression | 2.24 (1.48 – 3.40) | 1.4x10^-4^ | 2.20 (1.47 - 3.28) | 1.1x10^-4^ |
| Longest duration of depression | 1.46 (1.19 – 1.84) | 6.0x10^-4^ | 1.38 (1.14 - 1.69) | 1.4x10^-3^ |
| Number of episodes of depression | 1.43 (1.18 – 1.75) | 3.7x10^-4^ | 1.39 (1.16 - 1.68) | 5.2x10^-4^ |
| GAS depression | 0.47 (0.37 – 0.59) | 2.0x10^-10^ | 0.49 (0.39 - 0.60) | 7.2x10^-11^ |
| Depression onset first | 2.88 (1.59 – 5.47) | 7.1x10^-4^ | 2.70 (1.55 - 4.88) | 6.9x10^-4^ |

## Supplementary Table 5 Comparison of demographic, premorbid, and lifetime clinical characteristic results between schizoaffective bipolar-type and depressive-type.

Odds ratios, 95% confidence intervals and p-values are presented comparing schizoaffective disorder bipolar-type (SA-BP) to SA-D. Primary results in schizophrenia compared to SA-D are presented for reference.

| **Phenotype** | **SA-D vs SA-BP** | | **SA-D vs SCZ** | |
| --- | --- | --- | --- | --- |
|  | **OR (95% CI)** | **P-value** | **OR (95% CI)** | **P-value** |
| Female sex | 1.37 (0.82 - 2.28) | 0.23 | 3.19 (2.23 – 4.59) | 2.8x10^-10^ |
| Family history of schizophrenia | 1.33 (0.66 - 2.79) | 0.43 | 0.73 (0.45 – 1.15) | 0.18 |
| Family history of other psychiatric illness | 0.78 (0.45 - 1.36) | 0.39 | 1.50 (1.01 – 2.22) | 0.04 |
| Marital history | 0.93 (0.52 - 1.67) | 0.82 | 1.34 (0.88 – 2.01) | 0.16 |
| Number of children | 1.63 (1.14 - 2.43) | 0.01 | 1.34 (1.08 – 1.67) | 0.01 |
| Urbanicity | 0.63 (0.35 - 1.11) | 0.11 | 0.94 (0.63 – 1.40) | 0.77 |
| Premorbid social functioning | 1.23 (0.71 - 2.14) | 0.46 | 1.03 (0.70 – 1.50) | 0.88 |
| Obstetric complications | 1.12 (0.62 - 2.06) | 0.72 | 1.62 (1.03 – 2.50) | 0.03 |
| Educational attainment | 0.75 (0.63 - 0.88) | 7.4x10^-4^ | 1.02 (0.92 – 1.14) | 0.70 |
| Premorbid IQ | 0.71 (0.52 - 0.97) | 0.04 | 1.28 (1.04 – 1.60) | 0.02 |
| Childhood abuse | 1.61 (0.87 - 3.06) | 0.14 | 2.07 (1.35 – 3.17) | 7.9x10^-4^ |
| Course of disorder | 1.22 (0.93 - 1.62) | 0.16 | 0.81 (0.67 – 0.97) | 0.02 |
| Mode of onset | 1.08 (0.88 - 1.33) | 0.48 | 0.91 (0.79 – 1.05) | 0.18 |
| Antipsychotic response | 1.16 (0.66 -2.04) | 0.60 | 1.59 (1.08 – 2.35) | 0.02 |
| Cognition | 0.80 (0.64 - 0.99) | 0.04 | 1.20 (1.04 – 1.40) | 0.01 |
| Alcohol dependence | 1.04 (0.60 - 1.83) | 0.89 | 2.12 (1.41 – 3.20) | 3.2x10^-4^ |
| Cannabis dependence | 1.15 (0.62 - 2.16) | 0.66 | 1.18 (0.75 – 1.85) | 0.47 |
| Other substance dependence | 1.33 (0.70 - 2.59) | 0.39 | 1.26 (0.79 – 1.97) | 0.33 |
| Age at onset of psychosis | 1.31 (0.96 - 1.81) | 0.10 | 1.26 (1.03 – 1.54) | 0.02 |
| Number admissions for psychosis | 0.76 (0.52 - 1.02) | 0.10 | 0.97 (0.80 – 1.17) | 0.78 |
| Detained under the mental health act for psychosis | 0.69 (0.28 - 1.60) | 0.40 | 0.40 (0.22 – 0.75) | 3.2x10^-3^ |
| Number of episodes of psychosis | 0.81 (0.60 - 1.08) | 0.16 | 1.01 (0.81 – 1.22) | 0.94 |
| GAS psychosis | 1.59 (1.20 - 2.13) | 1.6x10^-3^ | 1.44 (1.20 – 1.72) | 5.8x10^-5^ |
| Positive symptoms | 0.99 (0.70 - 1.14) | 0.87 | 0.92 (0.84 – 1.01) | 0.09 |
| Disorganised symptoms | 0.74 (0.64 - 0.85) | 6.2x10^-5^ | 0.81 (0.72 – 0.91) | 5.3x10^-4^ |
| Diminished expressivity | 1.15 (1.03 - 1.30) | 0.02 | 1.03 (0.96 – 1.10) | 0.27 |
| Reduced motivation/pleasure | 1.05 (0.94 - 1.18) | 0.37 | 1.05 (0.97 – 1.13) | 0.42 |
| Age at first impairment from depression | 1.27 (0.92 - 1.81) | 0.16 | 0.98 (0.78 – 1.21) | 0.83 |
| Ever admitted for depression | 1.07 (0.60 - 1.93) | 0.81 | 2.24 (1.48 – 3.40) | 1.4x10^-4^ |
| Longest duration of depression | 1.86 (1.17 - 3.38) | 0.02 | 1.46 (1.19 – 1.84) | 6.0x10^-4^ |
| Number of episodes of depression | 1.18 (0.88 - 1.64) | 0.28 | 1.43 (1.18 – 1.75) | 3.7x10^-4^ |
| GAS depression | 0.62 (0.45 - 0.83) | 2.0x10^-3^ | 0.47 (0.37 – 0.59) | 2.0x10^-10^ |
| Depression onset first | 3.24 (1.50 - 7.18) | 3.1x10^-3^ | 2.88 (1.59 – 5.47) | 7.1x10^-4^ |

## Supplementary Table 6. Results of replication and meta-analysis.

Odds ratio with 95% confidence intervals and p-value are reported for the association between each variable and SA-D in the primary CardiffCOGS analysis, the replication in Cardiff Affected-sib and Cardiff F-series, and in a meta-analysis of all three samples.

| **Phenotype** | **CardiffCOGS** | | **Replication** | | **Meta-analysis** | |
| --- | --- | --- | --- | --- | --- | --- |
|  | **OR (95 %CI)** | **P-value** | **OR (95 %CI)** | **P-value** | **OR (95 %CI)** | **P-value** |
| Female sex | 3.19 (2.23 – 4.59) | 2.8x10^-10^ | 2.32 (1.21 - 4.44) | 0.01 | 2.96 (2.16 - 4.06) | 1.5x10^-11^ |
| Family history of other psychiatric illness | 1.50 (1.01 – 2.22) | 0.04 | 2.83 (1.31 - 6.13) | 0.01 | 1.71 (1.20 - 2.42) | 2.7x10^-3^ |
| Course of disorder | 0.81 (0.67 – 0.97) | 0.02 | 0.75 (0.51 - 1.11) | 0.16 | 0.80 (0.67 - 0.94) | 0.01 |
| Alcohol dependence | 2.12 (1.41 – 3.20) | 3.2x10^-4^ | 1.76 (0.87 - 3.57) | 0.12 | 2.03 (1.42 - 2.89) | 9.8x10^-5^ |
| Age at onset of psychosis | 1.26 (1.03 – 1.54) | 0.02 | 1.73 (1.26 - 2.37) | 7.4x10^-4^ | 1.38 (1.17 - 1.64) | 2.0x10^-4^ |

## Supplementary Table 7. Demographic and clinical characteristics in restricted sample univariable and multivariable models.

Odds ratios (OR) with 95% confidence intervals (CI) and p-values for the association between each characteristic and SA-D in the CardiffCOGS sample restricted to only those with complete data for the multivariable model. OR with 95% CI and p-values for the association between each characteristic and SA-D when analysed as part of a multivariable model. Table shading indicates the characteristics included in each model.

| **Phenotype** | **Schizophrenia sample size** | **SA-D sample size** | **Restricted univariable analysis** | | **Multivariable analysis** | |
| --- | --- | --- | --- | --- | --- | --- |
|  |  |  | **OR (95% CI)** | **P-value** | **OR (95% CI)** | **P-value** |
| Female sex | 219 | 54 | 2.97 (1.61 – 5.53) | 5.0x10^-4^ | 2.22 (1.14 – 4.36) | 0.02 |
| Family history of other psychiatric illness |  |  | 2.03 (1.08 – 3.84) | 0.03 | 1.77 (0.91 – 3.44) | 0.09 |
| Number of children |  |  | 1.10 (0.81 – 1.49) | 0.52 | 1.18 (0.85 – 1.62) | 0.32 |
| Obstetric complications |  |  | 1.88 (0.84 – 4.03) | 0.11 | 1.96 (0.84 – 4.41) | 0.11 |
| Premorbid IQ |  |  | 1.18 (0.86 – 1.65) | 0.31 | 1.16 (0.83 – 1.63) | 0.40 |
| Childhood abuse |  |  | 2.67 (1.33 – 5.30) | 0.01 | 2.80 (1.36 – 5.71) | 4.6x10^-3^ |
| Age at onset of psychosis |  |  | 1.12 (0.80 – 1.55) | 0.50 | 1.06 (0.75 – 1.49) | 0.72 |
| Course of disorder | 562 | 110 | 0.81 (0.65 – 1.00) | 0.04 | 0.86 (0.68 – 1.10) | 0.24 |
| Cognition |  |  | 1.14 (0.97 – 1.35) | 0.12 | 1.09 (0.91 – 1.30) | 0.35 |
| Alcohol dependence |  |  | 1.99 (1.26 – 3.13) | 2.8x10^-3^ | 2.08 (1.31 – 3.28) | 1.8x10^-3^ |
| Antipsychotic response |  |  | 1.53 (1.00 – 2.34) | 0.05 | 1.32 (0.81 – 2.14) | 0.26 |
| Detained under the mental health act for psychosis | 584 | 127 | 0.38 (0.21 – 0.72) | 2.4x10^-3^ | 0.55 (0.29 – 1.09) | 0.08 |
| GAS psychosis |  |  | 1.42 (1.17 – 1.73) | 4.0x10^-4^ | 1.31 (1.07 – 1.61) | 0.01 |
| Disorganised symptoms |  |  | 0.82 (0.72 – 0.93) | 2.3x10^-3^ | 0.84 (0.74 – 0.96) | 0.01 |
| Ever admitted for depression | 184 | 71 | 2.42 (1.31 – 4.46) | 4.6x10^-3^ | 1.65 (0.83 – 3.27) | 0.15 |
| Longest duration of depression |  |  | 1.31 (1.01 – 1.71) | 0.04 | 1.30 (0.96 – 1.78) | 0.10 |
| Number of episodes of depression |  |  | 2.03 (1.48 – 2.80) | 1.3x10^-5^ | 1.62 (1.14 – 2.32) | 0.01 |
| GAS depression |  |  | 0.39 (0.27 – 0.55) | 6.7x10^-7^ | 0.49 (0.33 – 0.72) | 4.8x10^-4^ |
| Depression onset first |  |  | 2.20 (1.14 – 4.47) | 0.02 | 1.27 (0.60 – 2.76) | 0.54 |

## Supplementary Table 8. Polygenic risk score results.

Results for all thresholds tested for schizophrenia and depression polygenic risk scores.

| **PRS** | **Threshold** | **OR (95% CI)** | **P-value** |
| --- | --- | --- | --- |
| Schizophrenia | 5x10^-8^ | 0.92 (0.75 – 1.14) | 0.45 |
|  | 1x10^-4^ | 0.82 (0.66 – 1.02) | 0.07 |
|  | 0.001 | 0.86 (0.69 – 1.06) | 0.16 |
|  | 0.05 | 0.94 (0.77 – 1.17) | 0.60 |
|  | 0.1 | 0.94 (0.76 – 1.16) | 0.55 |
|  | 0.5 | 0.89 (0.72 – 1.10) | 0.28 |
| Depression | 5x10^-8^ | 1.10 (0.89 – 1.36) | 0.39 |
|  | 1x10^-4^ | 1.17 (0.94 – 1.45) | 0.15 |
|  | 0.001 | 1.19 (0.96 – 1.48) | 0.12 |
|  | 0.05 | 1.26 (1.02 – 1.56) | 0.03 |
|  | 0.1 | 1.24 (1.00 – 1.54) | 0.05 |
|  | 0.5 | 1.31 (1.05 – 1.63) | 0.02 |
| Bipolar disorder | 5x10^-8^ | 0.94 (0.75 - 1.16) | 0.55 |
|  | 1x10^-4^ | 1.04 (0.83 - 1.30) | 0.73 |
|  | 0.001 | 1.07 (0.86 - 1.34) | 0.52 |
|  | 0.05 | 1.13 (0.91 - 1.42) | 0.27 |
|  | 0.1 | 1.18 (0.94 - 1.48) | 0.15 |
|  | 0.5 | 1.20 (0.95 - 1.52) | 0.13 |

References

1. Upthegrove, R. *et al.* Adverse childhood events and psychosis in bipolar affective disorder. *Br. J. Psychiatry* **206**, 191–197 (2015).
